# Circular RNA circ_001842 plays an oncogenic role in renal cell carcinoma by disrupting microRNA‐502‐5p‐mediated inhibition of SLC39A14

**DOI:** 10.1111/jcmm.15529

**Published:** 2020-07-30

**Authors:** Jiawei Zeng, Qian Feng, Yaodong Wang, Gang Xie, Yuanmeng Li, Yuwei Yang, Jiafu Feng

**Affiliations:** ^1^ Department of Clinical Laboratory Mianyang Central Hospital Mianyang China; ^2^ College of Medical Technology Chengdu University of Traditional Chinese Medicine Chengdu China; ^3^ Department of Urology Surgery Mianyang Central Hospital Mianyang China; ^4^ Department of Pathology Mianyang Central Hospital Mianyang China; ^5^ Department of Medical Laboratory Affiliated Hospital of Southwest Medical University Luzhou China

**Keywords:** circular RNA circ_001842, microRNA‐502‐5p, renal cell carcinoma, SLC39A14

## Abstract

Renal cell carcinoma (RCC) is a common urologic malignancy, and up to 30% of RCC patients present with locally advanced or metastatic disease at the time of initial diagnosis. Increasing evidence suggests that circular RNAs (circRNAs) serve as genomic regulatory molecules in various human cancers. Our initial in silico microarray‐based analysis identified that circRNA circ_001842 was highly expressed in RCC. Such up‐regulation of circ_001842 in RCC was experimentally validated in tissues and cell lines using RT‐qPCR. Thereafter, we attempted to identify the role of circ_001842 in the pathogenesis of RCC. Through a series of gain‐ and loss‐of function assays, cell biological functions were examined using colony formation assay, Transwell assay, annexin V‐FITC/PI‐labelled flow cytometry and scratch test. A high expression of circ_001842 in tissues was observed as associated with poor prognosis of RCC patients. circ_001842 was found to elevate SLC39A14 expression by binding to miR‐502‐5p, consequently resulting in augmented RCC cell proliferation, migration and invasion, as well as EMT in vitro and tumour growth in vivo. These observations imply the involvement of circ_001842 in RCC pathogenesis through a miR‐502‐5p‐dependent SLC39A14 mechanism, suggesting circ_001842 is a potential target for RCC treatment.

## 1 INTRODUCTION

An estimated 403 262 new cases of renal cancer and 175 098 related deaths have been reported worldwide in 2018.^1^ Renal cell carcinoma (RCC), a type of cancer derived from the renal epithelium, accounts for more than 90% of renal cancers.^2^ More than half of the RCC patients have localized tumours, which may be successfully treated by total or partial resection or destruction by surgery,^3^ and approximately 35% patients present with metastatic RCC or relapse following local therapy, which typically necessitates systemic treatment.^4^ Since the 2000s, significant advances in treating RCC have been achieved, which include drugs targeting vascular endothelial growth factor (VEGF) and mammalian target of rapamycin (mTOR) pathways.^5^ Despite progress in the therapeutics, RCC‐associated deaths are increasing in most developed countries,^6^ indicating a substantial need to expand therapeutic options. To identify putative drug targets, further understanding of the molecular underpinnings of tumorigenesis in RCC becomes essential.

Recently, a class of newly discovered non‐coding RNAs with covalent loop structure, named circular RNAs (circRNAs), have emerged as regulators in different biological processes.^7^, ^8^ Aberrations in circRNAs have been related to varied diseases such as human cancers, kidney diseases and cardiovascular diseases.^9^ In RCC, circPCNXL2 was found to advance cancer progression by up‐regulating ZEB2 through decoying miR‐153.^10^ Additionally, another study found an increased expression of circ‐ZNF609 in RCC and demonstrated that up‐regulated circ‐ZNF609 promoted RCC cell proliferation and invasion ability.^11^ Accumulating evidence has revealed that some circRNAs can regulate microRNAs (miRNAs) by functioning as miRNA sponges and play a significant role in transcriptional control.^12^ Using the CircInteractome database, in silico analysis predicted that circ_001842 can bind to some miRNAs including miR‐502‐5p. In general, miRNAs, small non‐coding RNAs with length of 18‐25 nucleotides, are implicated in the alteration and reprogramming of somatic cells, with the potential to target a number of molecules and regulate protein output.^13^ MiR‐502‐5p, in particular, has been reported as a protective miR in several human diseases, including osteoarthritis,^14^ breast cancer ^15^ and colon cancer.^16^ Several web‐available databases exploring miRNA‐mRNA interactions show that the gene ‘solute carrier family 39 member 14’ (SLC39A14) is a potential target of miR‐502‐5p. SLC39A14 is a member of the SLC39A transmembrane metal transporter family.^17^ SLC39A14 has been found up‐regulated in gastric cancer and thus has been considered as a prognostic biomarker.^18^ In contrast, in prostate cancer, low expression of SLC39A14 has been associated with the aggressiveness of malignant tumour and tumour progression.^19^ Considering that such opposing findings suggest SLC39A14 may exhibit variable expression patterns and effects on different human cancers, its role in RCC needs to be elucidated. The present study aims to determine the role played by the circ_001842/miR‐502‐5p/SLC39A14 regulatory network in RCC, in a bid to enhance the understanding of the mechanisms underlying RCC carcinogenesis.

## 2 MATERIALS AND METHODS

### 2.1 Ethics statement

A prospective observational study was designed, which included all patients with renal tumours treated surgically at the Department of Urology, Mianyang Central Hospital, Affiliated to Southwest Medical University, during the study period. This study protocol was approved by the Human Ethics Committees Review Board of Mianyang Central Hospital, Affiliated to Southwest Medical University (S2014048, S2018085), and all procedures were conducted in strict accordance with the Declaration of Helsinki. Signed informed consent was obtained from all participants prior to the study. All animal experiment protocols were authorized and approved by the Animal Ethics Committee of Mianyang Central Hospital, Affiliated to Southwest Medical University. These experiments strictly followed the principle of completion of experiments by including the minimum number of animals required and minimizing the pain of the experimental animals.

### 2.2 Study participants

A total of 97 RCC patients (63 males and 34 females, aged 35‐76 years with a mean age of 55.37 ± 8.30 years) who underwent radical or partial nephrectomy at the Department of Urology of Mianyang Central Hospital, Affiliated to Southwest Medical University from January 2015 to June 2019, were enrolled in the study. No patients received neoadjuvant chemotherapy and immunotherapy prior to surgery. The baseline demographic and clinical characteristics of the patients are shown in Table 1. Urine and blood samples were taken in the morning before the surgery. The urine albumin‐creatinine ratio (UACR) was calculated. The estimated glomerular filtration rate (eGFR) was calculated using serum Cr and cystatin C (CysC) levels by applying the CKD‐EPI Equation ^20^ All patients underwent radical or partial nephrectomy at the Department of Urology of Mianyang Central Hospital, Affiliated to Southwest Medical University, and none of them received neoadjuvant chemotherapy and immunotherapy prior to surgery. During surgery, kidney tissues were immediately bisected, and non‐neoplastic cortical tissue and normal paracancerous tissues visible to the naked eye were excised with a scalpel. Excised tissue samples were immediately placed on ice, frozen in liquid nitrogen and stored at − 80°C for further experiments. All excised tissues were examined by trained pathologists, tumours were staged according to the tumour node metastasis (TNM) classification and criteria of World Health Organization (WHO), and tumour grade was assigned in accordance with the WHO criteria. In addition, human RCC cell lines, Caki‐1, 769‐P, OSRC‐2 and La ribonucleoprotein domain family member 6 (ACHN) (ATCC, Manassas, VA, USA), and human normal renal epithelial cell line hexokinase 2 (HK‐2) (ATCC, USA) were purchased. Furthermore, a total of 32 male nude mice aged 4‐5 weeks weighing 16‐20 g were purchased from Shanghai SLAC Laboratory Animals Co., Ltd. (Shanghai, China).

**TABLE 1:** Association between RNA hsa_circ_001842 expression and clinicopathological characteristics in 97 renal cell carcinoma patients

Variable	hsa_circ_001842 expression		χ^2^/*t*	*P* value
	Low expression	High expression		
	(n = 49)	(n = 48)		
Age (years)				
<60	30	28	0.084	0.772
≥60	19	20		
Gender				
Male	29	34	1.445	0.229
Female	20	14		
Pathological grade				
I–II	24	11	7.142	0.008
III–IV	25	37		
Lymph node metastasis				
NO‐N1	23	12	5.060	0.025
N2‐NX	26	36		
Distant metastasis				
Absent	27	16	4.656	0.031
Present	22	32		
Tumour size				
T1‐T2	30	13	11.450	0.001
T3‐T4	19	35		
TNM stage				
I‐II	38	12	26.81	<0.001
IIIa	11	36		
UACR (mg/g)				
<30	31	31	0.018	0.893
≥30	18	17		
eGFR (mL/min/1.73 m^2^)				
≥60	36	37	0.170	0.680
<60	13	11		
Cr (μmol/L)				
<82.1 (F)/97.0 (M)	37	39	0.471	0.493
≥82.1 (F)/97.0 (M)	12	9		
CysC (mg/L)				
<1.09	37	34	0.270	0.603
≥1.09	12	14		

Note

The difference in gender was compared by chi‐squared test and that in others by Welch t test. CysC, cystatin C; TNM, tumour node metastasis; UACR, urinary albumin‐to‐creatinine ratio; SCr, serum creatinine; eGFR, estimated glomerular filtration rate; eGFR = 78.64 × CysC^‐0.964^.

### 2.3 Reverse transcription‐quantitative polymerase chain reaction (RT‐qPCR)

Total RNA was extracted with TRIzol and reverse‐transcribed into complementary DNA (cDNA) using the ReverTra Ace^®^ qPCR RT Master Mix with gDNA Remover Kit (TOYOBO, Japan). The cDNA samples were then amplified using CFX96™ Real‐Time System (Bio‐Rad, Hercules, CA, USA), and RT‐qPCR was carried out using the 2 × RealStar Green Mixture (GenStar, Hangzhou, China) kit, in accordance with the kit instructions. The primers for circ_001842, miR‐502‐5p, SLC39A14, E‐cadherin, N‐cadherin, matrix metalloproteinase‐9 (MMP‐9), glyceraldehyde‐3‐phosphate dehydrogenase (GAPDH) (for mRNA normalization) and U6 (for miRNA normalization) were synthesized (Shanghai Biotech) (shown in Table 2). mRNA and miRNA relative expression levels were assessed using the 2^(‐ΔΔCt)^ relative quantification method in which ΔCt = Ct _(target)_ ‐ Ct _(GAPDH/U6)._

**TABLE 2:** Primer sequences for RT‐qPCR

Target of interest	Sequence
hsa_circ_001842‐F	5’‐AATGCTGAAAACTGCTGAGAGAA‐3’
hsa_circ_001842‐R	5’‐TTGAGAAAACGAGTGCTTTGG‐3’
miR‐502‐5p‐F	5’‐ATCCTTGCTATCTGGGTGCTA‐3’
miR‐502‐5p‐R (RTQ‐UNIr)	5’‐CGAATTCTAGAGCTCGAGGCAGGCGACATGGCTGGCTAGTTAAGCTTGGTACCGAGCTCGGATCCACTAGTCC(T)‐3’
SLC39A14‐F	5’‐GCTTCCCTCCAAGAAGGACC‐3’
SLC39A14‐R	5’‐TAGCAAGCACTCTGGGAAGC‐3’
E‐cadherin‐F	5’‐CCCTCGACACCCGATTCAAA‐3’
E‐cadherin‐R	5’‐TCTGTAGGTGGAGTCCCAGG‐3’
N‐cadherin‐F	5’‐CTGTCCCCGGCGTCTTC‐3’
N‐cadherin‐R	5’‐GATGGCGCTCCCCAAGAG‐3’
MMP‐9‐F	5’‐CCTGGGCAGATTCCAAACCT‐3’
MMP‐9‐R	5’‐GTACACGCGAGTGAAGGTGA‐3’
U6‐F	5’‐CTCGCTTCGGCAGCACA‐3’
U6‐R	5’‐AACGCTTCACGAATTTGCGT‐3’
GAPDH‐F	5’‐GCAAACAAGCAGGATCAGCAA‐3’
GAPDH‐R	5’‐CCCATCCTTGCCCTTGGTAA‐3’

Note

RT‐qPCR, reverse transcription‐quantitative polymerase chain reaction; F, forward; R, reverse.

### 2.4 RNase R tolerance test

Primers were designed by Yunxu Biotechnology Co., Ltd. (Shanghai, China) and synthesized by Beijing Genomics Institute (Beijing, China). The specificity of the PCR products was verified by RT‐qPCR and PCR gel electrophoresis. RNase R was used to treat the total RNA, and RT‐qPCR was used to determine the relative expression levels of circ_001842 before and after treatment.

### 2.5 Cell transfection

Caki‐1 cells were transfected with the following plasmids: overexpression (oe)‐circ_001842, oe‐SLC39A14, short hairpin RNA (sh)‐circ_001842, sh‐SLC39A14, mimic negative control (NC), sh‐NC, miR‐502‐5p mimic, miR‐502‐5p inhibitor alone or in combination. All plasmids were supplied by Sino Biological (Beijing, China) and transfected into Caki‐1 cells using Lipofectamine 2000 reagents (Invitrogen, Carlsbad, CA, USA). After 48 hours of transfection, cells were retrieved for further analysis.

### 2.6 Colony formation assay

Caki‐1 cells were seeded in 6‐well plates at a gradient density of 50, 100 and 200 cells per dish. The medium was placed in a 5% CO_2_ incubator at 37°C for 2‐3 weeks. When colonies were visible without a microscope, they were fixed with 5 mL 4% paraformaldehyde and stained with GIMSA solution (Invitrogen, USA). The staining solution was then washed with running water followed by air‐drying. The number of cell clones was observed under an inverted optical microscope and counted. The colony formation rate was calculated based on the formula: colony formation rate = number of clones formed/number of cells incubated.

### 2.7 Flow cytometry

Cell apoptosis was detected using the Annexin V‐Fluorescein Isothiocyanate (FITC) Apoptosis Detection Kit (Becton Dickinson; Franklin Lakes, NJ, USA). In brief, the cells were seeded in 6‐well plates at a density of 2 × 10^5^ cells/well and transfected. After 72 hours, the collected cell suspension was centrifuged at 800 g, and the supernatant was discarded. The cells were washed with phosphate‐buffered saline (PBS), resuspended in 500 μL binding buffer, added with 5 μL FITC and 5 μL propidium iodide (PI) in the dark and incubated for 15 minutes. Thereafter, cell apoptosis was measured by flow cytometry (FACSCalibur, Beckman Coulter Inc, Brea, CA, USA).

### 2.8 Immunoblotting

Total protein was extracted using radioimmunoprecipitation assay (RIPA) buffer (Beyotime Institute of Biotechnology Co., Ltd., Shanghai, China). Total protein concentration was detected using a Bicinchoninic Acid (BCA) Protein Assay Kit (Pierce Biotechnology Inc, Rockford, IL, USA). Protein samples were separated by sodium dodecyl sulphate‐polyacrylamide gel electrophoresis (SDS‐PAGE) and transferred to polyvinylidene fluoride (PVDF) membranes. The membranes were blocked with 5% milk powder for 1 hours and incubated with the primary antibody against SLC39A14 (Abcam Inc, Cambridge, UK) at 4℃ overnight. After being rinsed with PBS containing 0.1% Tween‐20 (PBST), the membrane was incubated with horseradish peroxidase (HRP)‐labelled immunoglobulin G (IgG) (Santa Cruz, USA). The immunocomplexes on the membrane were visualized with enhanced chemiluminescence (ECL) reagent, and band intensities were quantified using the ImageJ2x software. The ratio of the grey value of protein band to that of the internal reference band, mouse antibody against GAPDH (Santa Cruz, CA, USA), was used as the relative expression level of the protein.

### 2.9 Fluorescence in situ hybridization (FISH) assays

The subcellular localization of circ_001842 and miR‐502‐5p in Caki‐1 cells was identified by FISH assay following the instructions of Ribo™ lncRNA FISH Probe Mix (Red) (Guangzhou RiboBio Biotechnology Co., Ltd., Guangzhou, China). Caki‐1 cells were seeded on a coverslip in a 6‐well plate and then incubated for 24 hours when cell confluence reached about 80%. The cells were fixed with 4% paraformaldehyde (1 mL) and treated with proteinase K (2 μg/mL), glycine and acetamidine reagent. Next, they were incubated with 250 μL pre‐hybridization solution for 1 hours at 42°C, followed by incubation with 250 μL hybridization solution containing probes (300 ng/mL) and staining with PBST‐diluted 4′6‐diamidino‐2‐phenylindole (DAPI) (1:800) for 5 minutes. Finally, the slides were washed with PBST and sealed with antifluorescence quenching blocking solution. The cells were observed and photographed in 5 randomly selected visual fields under a fluorescence microscope (Olympus, Tokyo, Japan).

### 2.10 RNA pull‐down assay

The biotin probe was designed to bind the junction region of circ_001842. Approximately 1 × 10^7^ cells were washed in pre‐chilled PBS, lysed and incubated with 3 μg biotin probes. The cell lysates were incubated with streptavidin magnetic beads (Life technologies corporation, Gaithersburg, MD, USA) for 4 hours to pull down the biotin‐conjugated RNA complexes. The magnetic beads were then washed 5 times with lysis buffer, and bound RNA was extracted using TRIzol reagent. Enrichment was analysed by RT‐qPCR.

### 2.11 Dual‐luciferase reporter assay

The predicted binding site fragment and mutant fragment of circ_001842 and miR‐502‐5p were each inserted into the respective vector, namely reporter plasmid circ_001842‐wild type (WT) and circ_001842‐mutant type (MUT). In order to determine whether circ_001842 could bind to miR‐502‐5p, NC and miR‐502‐5p mimic were cotransfected into 293T cells (Oulu Biotechnology, China) with circ_001842 luciferase reporter plasmid, with Renilla luciferase set as the loading control. Following 48 hours of transfection, the cells were collected and lysed. Luciferase Reporter Assay Kit (K801‐200, BioVision, Milpitas, CA, USA) was used according to kit instructions, and a Dual‐Luciferase Reporter Analysis System (Promega, Madison, WI, USA) was used to perform the assay. Relative luciferase activity was quantified as the ratio of firefly luciferase relative light unit (RLU) to Renilla luciferase RLU.

The predicted binding site fragment and mutant fragment of 3’‐untranslated region (3'‐UTR) and miR‐502‐5p of SLC39A14 were each inserted into the respective vector, namely reporter plasmids SLC39A14‐WT and SLC39A14‐MUT. NC and miR‐502‐5p mimic and SLC39A14 mRNA luciferase reporter plasmid were cotransfected to determine whether SLC39A14 bound to miR‐502‐5p. The specific steps were the same as described earlier.

### 2.12 Transwell invasion assay

The membrane of the Transwell apical chamber was coated with pre‐cooled Matrigel. Caki‐1 cells were resuspended in a serum‐free Opti‐MEM I medium (Invitrogen Inc, CA, USA) and adjusted to a density of 3 × 10^4^ cells/mL. Then, 100 μL cell suspension was inoculated, while 600 μL Roswell Park Memorial Institute (RPMI) 1640 medium with 10% foetal bovine serum (FBS) was added into the lower chambers. After 24 hours of incubation, the cells were fixed with 4% paraformaldehyde and then stained with 0.05% gentian violet for 5 minutes. Migrated cells were photographed and counted using randomly selected 5 visual fields under an inverted microscope.

### 2.13 Scratch test

Caki‐1 cells were seeded into 6‐well plates at a density of 5 × 10^5^ cells/well. When the cells were fully adherent, a 2‐mm scratch was made through the cell monolayer in the middle of each well. Next, the cells were cultured in serum‐starved medium for 24 hours. The cells were observed and photographed at 0 hour and 24 hours. The scratch healing ratio was calculated using Image‐Pro Plus 6.0.

### 2.14 Xenograft tumour in nude mice

The mice (4‐5 weeks old, 16‐20 g, Shanghai SLAC Laboratory Animal Co. Ltd., Shanghai, China) were randomly divided into 2 groups (n = 16 for each group): oe‐NC and oe‐circ_001842 (mice inoculated with cells transfected with oe‐NC and oe‐circ_001842 plasmids, respectively). Caki‐1 cells infected with GFPSpark® lentivirus (Sino biological, USA) were dispersed into a cell suspension at a density of 1 × 10^6^ cells/mL. The mice were anaesthetized with ether and then inoculated subcutaneously on the back of the right hind leg with 200 μL of the cell suspension. The short diameter (a) and long diameter (b) of the tumours were measured and recorded every 7 days. The volume of the tumours was calculated with the following formula: tumour volume = ab^2^/2. Four weeks later, all mice were killed. The tumours were exfoliated and weighed using a balance.

### 2.15 Immunohistochemistry

The paraffin‐embedded tumour tissues were sliced into 5‐μm sections, followed by dewaxing with xylene and dehydration with gradient alcohol. After microwave‐stimulated antigen retrieval, the sections were blocked with 1% bovine serum albumin (BSA) sealing reagent. Next, the sections were incubated with the following primary antibodies: SLC39A14 antibody (1:500, Abcam Inc, Cambridge, UK), E‐cadherin antibody (1:300, Abcam Inc, Cambridge, UK) and N‐cadherin antibody (1:300, Abcam Inc, Cambridge, UK). The samples were washed with PBS and incubated with HRP‐labelled anti‐rabbit secondary antibody (1:100, Boster Biological Engineering Co., Ltd., Wuhan, China) at room temperature for 1 hours 3, 3'‐diaminobenzidine (DAB) (Boster Biological Engineering Co., Ltd., Wuhan, China) was used to develop the sections, and haematoxylin was used to stain the nuclei. The sections were then observed and photographed under an optical microscope.

### 2.16 Statistical analysis

Data were analysed using SPSS 21.0 software (IBM Corp. Armonk, NY, USA). Measurement data were presented as mean ± standard deviation. Normal distribution and variance homogeneity test were carried out. If the data conformed to the normal distribution and homogeneity, within‐group comparison was performed using paired t test and between‐group comparison was performed using t test. Comparisons among multiple groups were performed by one‐way analysis of variance (ANOVA), followed by Tukey's post hoc test. Data from different time‐points were compared using repeated‐measures ANOVA. Pearson's correlation analysis was applied to analyse the correlation of circ_001842 with miR‐502‐5p, and miR‐502‐5p with SLC39A14. The Kaplan‐Meier method was applied to calculate the survival rate of patients, and univariate analysis was performed by log‐rank test. A value of *P* < .05 indicated statistical significance.

## 3 RESULTS

### 3.1 circ_001842 is highly expressed in RCC

**FIGURE 1:**
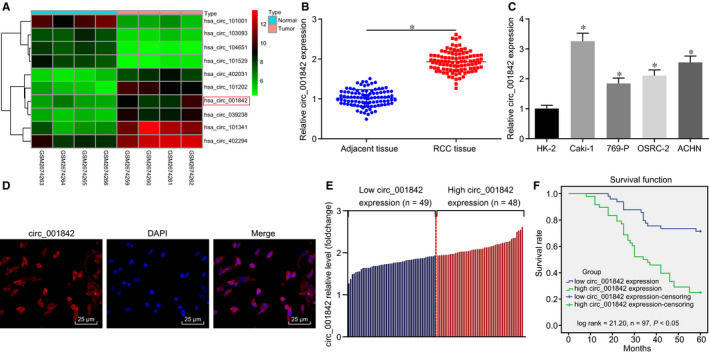
High expression of circ_001842 in RCC tissues and cells. A, The heatmap showing the top 10 differentially expressed circRNAs. The x‐axis indicates the sample number, and the y‐axis refers to the names of circRNA; the right upper histogram indicates colour gradation; each rectangle in the figure corresponds to the expression of a circRNA in a sample. B, RT‐qPCR analysis of circ_001842 expression in 97 RCC and normal tissues, *P < .05 vs the normal tissues. C, RT‐qPCR analysis of circ_001842 expression in human normal renal epithelial cell line HK‐2 and four human RCC cell lines including Caki‐1, 769‐P, OSRC‐2 and ACHN, *P < .05 vs the normal tissues. D, Subcellular localization of circ_001842 in Caki‐1 cells detected by FISH assay, scale bar = 25 μm. E, Based on the median expression level of circ_001842 in RCC, the patients were divided into two groups: circ_001842 high expression (n = 48) and low expression (n = 49). F, Kaplan‐Meier single‐factor survival analysis (log‐rank test) performed based on circ_001842 expression categories. Data (mean ± SD) from three independent experiments; comparisons between two groups were analysed using t test and those between multiple groups were analysed using one‐way ANOVA, followed by Tukey's post hoc test

The gene expression data set GSE100186 was downloaded from the Gene Expression Omnibus (GEO) (https://www.ncbi.nlm.nih.gov/geo/). Differential expression analysis of circRNAs between RCC tissues (n = 4) and normal tissues (n = 4) was performed using the ‘Limma’ package in the R statistical environment. A heatmap (Figure 1A) showing the expression of the first 10 differentially expressed circRNAs revealed that circ_001842 was elevated in RCC tissues as compared with normal tissues. Next, we found that circ_001842 was an alias of circ_0000328 in the circBase database. The circ_001842 primer was subjected to PCR for gel extraction and sequencing, and the resulting Sanger sequence is shown in Figure S1. This sequence was our research object circ_001842. RT‐qPCR demonstrated that the expression of circ_001842 did not decrease significantly after RNase R treatment, whereas linear circ_001842 expression normalized to GAPDH was decreased significantly after RNase R treatment (Figure S2), suggesting that circ_001842 was resistant to RNase R.

The expression of circ_001842 in 97 RCC and normal tissues was further analysed by RT‐qPCR, and the results showed that expression of circ_001842 in RCC tissues was significantly increased (*P* < .05) (Figure 1B). Then, RT‐qPCR was used to determine the circ_001842 expression levels in human normal renal epithelial cell line HK‐2 and four human RCC cell lines: Caki‐1, 769‐P, OSRC‐2 and ACHN. The results showed significantly higher circ_001842 expression in the four human RCC cell lines than that in the HK‐2 cell line (*P* < .05), and the highest expression of circ_001842 was found in the Caki‐1 cell lines (Figure 1C). Therefore, Caki‐1 cells were selected for subsequent experiments. In addition, circ_001842 was found to be expressed in the cytoplasm of Caki‐1 cells using FISH (Figure 1D). Ninety‐seven patients with RCC were divided into high circ_001842 expression (n = 48) and low circ_001842 expression (n = 49) based on a cut‐off using the median of circ_001842 expression level (Figure 1E). The patient survival rate among the group with high circ_001842 expression was found to be lower than those with low circ_001842 expression level (*P* < .05) (Figure 1F), indicating a positive correlation between circ_001842 and the degree of RCC. These data suggested that circ_001842 expression was increased in commercial renal cancer lines and RCC tissues, and thus, it might play a relevant regulatory role in RCC.

### 3.2 Silencing circ_001842 impedes RCC cell proliferation, migration and invasion in vitro

**FIGURE 2:**
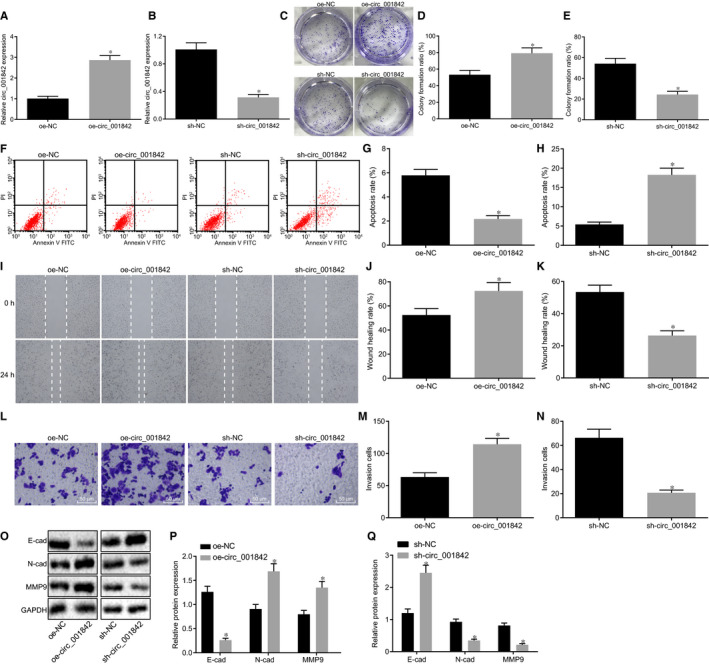
Silencing circ_001842 impedes the proliferation, migration and invasion of RCC cells in vitro. A, circ_001842 expression in Caki‐1 cells treated with oe‐NC and oe‐circ_001842 determined by RT‐qPCR. B, circ_001842 expression in Caki‐1 cells treated with sh‐NC and sh‐circ_001842 determined by RT‐qPCR. C‐E, Colony formation assay for cell proliferation in Caki‐1 cells after overexpression or silencing of circ_001842. F‐H, Apoptosis of Caki‐1 cells after overexpression or silencing of circ_001842 detected by flow cytometry. I‐K, Cell migration of Caki‐1 cells after overexpression or silencing of circ_001842 detected by scratch test. L‐N, Cell invasion after overexpression or silencing of circ_001842 in Caki‐1 cells measured by Transwell assay, scale bar = 50 μm. O‐Q, Western blot analysis of EMT‐related proteins E‐cadherin, N‐cadherin and MMP‐9 in Caki‐1 cells after overexpression or silencing of circ_001842. Data (mean ± standard error) from three independent experiments were compared between two groups using t test. *P < .05 vs the normal tissues

To further clarify the possible effect of circ_001842 on RCC, we constructed the overexpression and silencing sequences of circ_001842 and transfected them into Caki‐1 cells. Firstly, RT‐qPCR results showed that circ_001842 expression was increased after transfection of oe‐circ_001842 (*P* < .05, Figure 2A), whereas after transfection of sh‐circ_001842, the expression of circ_001842 was significantly decreased (*P* < .05, Figure 2B).Compared with NC, overexpression of circ_001842 in Caki‐1 cells notably enhanced cell proliferation ability (Figure 2C and D), reduced the apoptosis (Figure 2F and G) and increased the migration and invasion of Caki‐1 cells (*P* < .05, Figure 2I, J, L, and M). Versus controls, silencing circ_001842 in Caki‐1 cells reduced the cell proliferation, migration and invasion ability (Figure 2C, E, I, K, L, and N), but increased the level of apoptosis (Figure 2F, and H). Immunoblotting was used for detection of epithelial‐mesenchymal transition (EMT)‐related proteins E‐cadherin, N‐cadherin and MMP‐9 expression after overexpression or silencing of circ_001842 in Caki‐1 cells. The results displayed that E‐cadherin expression was decreased while N‐cadherin and MMP‐9 expression was increased after circ_001842 overexpression, which promoted EMT (Figure 2O, P). Upon silencing circ_001842, E‐cadherin expression was increased, while N‐cadherin and MMP‐9 expression was decreased, which suggested inhibited EMT (Figure 2O, and Q). These results indicated that circ_001842 promoted the proliferation, migration and invasion of Caki‐1 cells and the process of EMT in vitro.

### 3.3 circ_001842 competitively binds to miR‐502‐5p in RCC cells

**FIGURE 3:**
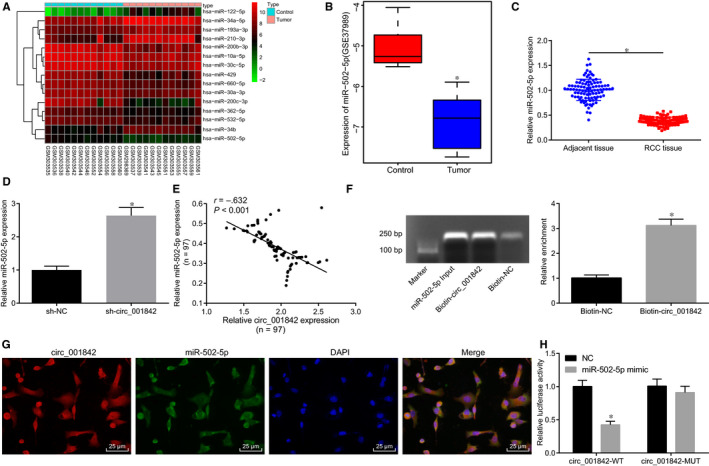
circ_001842 binds to miR‐502‐5p in vitro. A, The heatmap of the top 15 miRNAs in the GSE12105 data set. The x‐axis indicates the sample number, and the y‐axis refers to the names of miRNA; the right upper histogram indicates colour gradation; each rectangle in the figure corresponds to the expression of a miRNA in a sample. B, Expression of miR‐502‐5p in the GSE37989 data set. C, RT‐qPCR analysis of miR‐502‐5p expression in 97 RCC and normal tissues. D, The expression of miR‐502‐5p in Caki‐1 cells treated with sh‐NC and sh‐circ_001842 determined by RT‐qPCR. E, Pearson's correlation analysis of relative expression of circ_001842 and miR‐502‐5p in RCC. F, circ_001842 specific enrichment of miR‐502‐5p detected by RNA pull‐down assay after pull‐down products were amplified and subjected to agarose gel electrophoresis. G, Subcellular localization of circ_001842 in cells detected by FISH, scale bar = 25 μm. H, The binding between circ_001842 and miR‐502‐5p detected by dual‐luciferase reporter assay. Data (mean ± SD) from three cell experiments were compared between two paired groups using paired t test, and data from multiple groups were analysed using one‐way ANOVA, followed by Tukey's post hoc test. n = 97. *P < .05 vs the adjacent tissues or normal tissues

Previous FISH results indicated that circ_001842 was localized in the cytoplasm. To further explore the mechanism of circ_001842 in RCC, the CircInteractome database (https://circinteractome.nia.nih.gov/) was utilized to predict miRNAs that may bind to circ_001842. Eight such miRNAs were found: hsa‐miR‐1248, hsa‐miR‐432, hsa‐miR‐502‐5p, hsa‐miR‐526b, hsa‐miR‐577, hsa‐miR‐622, hsa‐miR‐640 and hsa‐miR‐885‐3p. Based on the differential analysis of GSE12105 and GSE37989 data sets, the heatmap of the top 15 miRNAs in GSE12105 was drawn (Figure 3A). Hsa‐miR‐502‐5p was down‐regulated in RCC tissues, and miR‐502‐5p was also poorly expressed in the GSE37989 data set (Figure 3B). It was speculated that circ_001842 might bind to miR‐502‐5p in RCC. The expression of miR‐502‐5p in 97 RCC and normal tissues was next analysed by RT‐qPCR, revealing down‐regulated miR‐502‐5p expression in RCC tissues (*P* < .05, Figure 3C). Upon inhibiting the expression of circ_001842 in Caki‐1 cells, the expression of miR‐502‐5p was increased (*P* < .05, Figure 3D), indicating that the expression pattern of miR‐502‐5p and circ_001842 was opposite in RCC and Caki‐1 cells (Figure 3E). In the pull‐down experiment, specific enrichment of miR‐502‐5p was detected in the beads pulled down by circ_001842 versus controls (Figure 3F). circ_001842 and miR‐502‐5p were found colocalized in the cytoplasm of Caki‐1 cells by FISH (Figure 3G). Thereafter, bioinformatics analysis predicted the existence of a binding site between circ_001842 and miR‐502‐5p, which was further confirmed by dual‐luciferase reporter assay. The results showed that circ_001842‐WT had a lower luciferase activity in cells transfected with miR‐502‐5p mimic (*P* < .05), while circ_001842‐MUT exhibited no significant changes (*P*> .05) (Figure 3H). The data collected indicated that circ_001842 could directly bind to miR‐502‐5p.

### 3.4 circ_001842 up‐regulates the expression of SLC39A14 by binding to miR‐502‐5p

**FIGURE 4:**
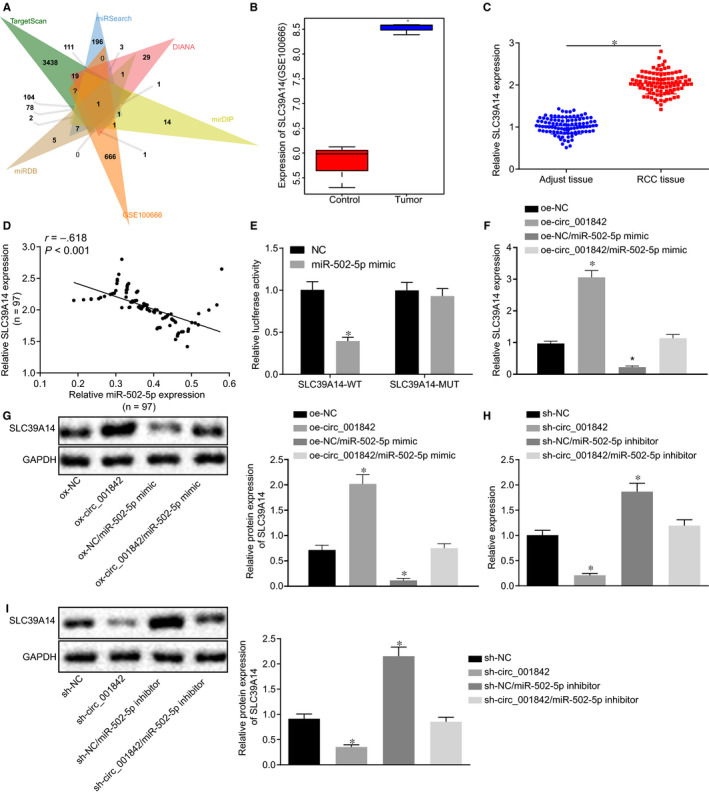
circ_001842 up‐regulates the expression of SLC39A14 by binding to miR‐502‐5p. A, predicted target genes of miR‐502‐5p using the TargetScan, miRSearch, DIANA, mirDIP and miRDB databases and differentially expressed genes obtained from the GSE100666 data set. B, SLC39A14 expression in the GSE100666 data set. C, SLC39A14 expression in RCC and normal tissues detected by RT‐qPCR, n = 97. D, Correlation analysis of the relative expression of miR‐502‐5p and SLC39A14 in RCC, n = 97. E, The binding between miR‐502‐5p and SLC39A14 detected by dual‐luciferase reporter assay. F‐G, The expression of SLC39A14 in Caki‐1 cells treated with oe‐NC, oe‐circ_001842, both oe‐NC and miR‐502‐5p mimic, and both oe‐circ_001842 and miR‐502‐5p mimic, determined by RT‐qPCR and Western blot analysis, respectively (*P* < .05). H‐I, The mRNA and protein expression of SLC39A14 in Caki‐1 cells transfected with sh‐NC, sh‐circ_001842, sh‐NC and miR‐502‐5p inhibitor and sh‐circ_001842 and miR‐502‐5p inhibitor, respectively, determined by RT‐qPCR and Western blot analysis. Data (mean ± SD) from three independent experiments were compared between two groups using unpaired t test and data from multiple groups were compared using one‐way ANOVA, followed by Tukey's post hoc test. *P < .05 vs the cells without treatment

The potential target genes of miR‐502‐5p were predicted using the TargetScan (http://www.targetscan.org/vert_71/), miRSearch (http://www.exiqon.com/microrna‐target‐prediction), DIANA (http://diana.imis.athena‐innovation.gr/DianaTools/index.php?r = microT_CDS/index), mirDIP (http://ophid.utoronto.ca/mirDIP/) and miRDB databases (http://www.mirdb.org/). The predicted results were compared with the differentially expressed genes obtained from the GSE100666 data set using a Venn diagram (http://jvenn.toulouse.inra.fr/app/example.html) (Figure 4A). There was only one overlapping gene SLC39A14, which was highly expressed in RCC tissues in the GSE100666 data set (Figure 4B). It was thus speculated that miR‐502‐5p might target SLC39A14 in RCC. The expression of SLC39A14 in 97 RCC and normal tissues analysed by RT‐qPCR was found to be significantly enhanced in RCC (*P* < .05, Figure 4C), whereas the expression of miR‐502‐5p showed an opposite trend (*P* < .05, Figure 4D). The subsequent results revealed that in cells cotransfected with miR‐502‐5p mimic and SLC39A14‐WT, the luciferase activity was decreased (*P* < .05) (Figure 4E), indicating that miR‐502‐5p bound to SLC39A14. Caki‐1 cells were then transfected with oe‐circ_001842, miR‐502‐5p mimic or both. RT‐qPCR and immunoblotting showed that versus oe‐NC transfection, SLC39A14 mRNA and protein expression was increased following transfection with oe‐circ_001842, but it was decreased upon transfection with oe‐NC/miR‐502‐5p mimic (all *P* < .05). SLC39A14 mRNA and protein expression in cells cotransfected with oe‐circ_001842 and miR‐502‐5p mimic was higher than that in cells cotransfected with oe‐NC and miR‐502‐5p mimic, while lower than that in cells cotransfected with oe‐circ_001842 (Figure 4F and G). Caki‐1 cells were transfected with sh‐circ_001842, miR‐502‐5p inhibitor or both. The results of RT‐qPCR and immunoblotting demonstrated that SLC39A14 mRNA and protein expression was reduced in the absence of circ_001842, but increased upon miR‐502‐5p inhibitor treatment (all *P* < .05). It was suggested that SLC39A14 expression in cells cotransfected with sh‐circ_001842 and miR‐502‐5p inhibitor was higher than that in cells treated with sh‐circ_001842, yet lower than that in cells treated with miR‐502‐5p inhibitor (Figure 4H and I). These results indicated that circ_001842 up‐regulated the expression of SLC39A14 in tumour cells by binding to miR‐502‐5p.

### 3.5 circ_001842 silencing inhibits RCC cell proliferation, migration, invasion and EMT via miR‐502‐5p‐mediated SLC39A14 inhibition

**FIGURE 5:**
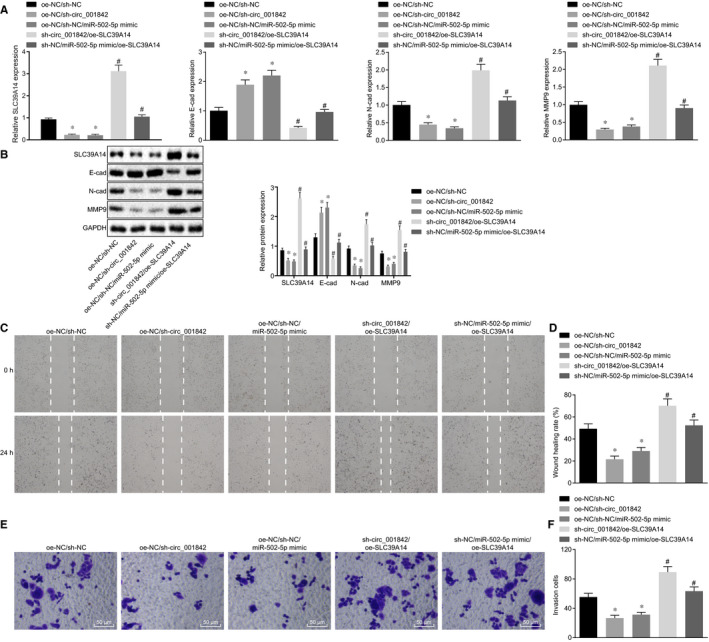
circ_001842/miR‐502‐5p regulates the proliferation, migration and EMT of renal cancer cells via SLC39A14. A‐B, RT‐qPCR and Western blot analysis of EMT‐related proteins SLC39A14, E‐cadherin, N‐cadherin and MMP‐9 in Caki‐1 cells treated with oe‐NC/sh‐NC, oe‐NC/sh‐circ_001842, oe‐NC/sh‐NC/miR‐502‐5p mimic, sh‐circ_001842/oe‐SLC39A14 and sh‐NC/miR‐502‐5p mimic/oe‐SLC39A14. *P < .05 vs the cells treated with sh‐NC, #*P* < .05 vs the cells treated with single plasmids. C‐D, The migration ability of Caki‐1 cells in each group detected by scratch test, *P < .05 vs the cells treated with sh‐NC, #*P* < .05 vs the cells treated with single plasmids. E‐F, The invasion ability of Caki‐1 cells in each group detected by Transwell invasion assay, scale bar = 50 μm. Data (mean ± SD) from three cell experiments were compared between multiple groups using one‐way ANOVA followed by Tukey's post hoc test. *P < .05 vs the cells treated with sh‐NC, and #*P* < .05 vs the cells treated with single plasmids

After silencing circ_001842 or overexpressing miR‐502‐5p in Caki‐1 cell lines, the expression of EMT‐related proteins E‐cadherin, N‐cadherin and MMP‐9 was determined by RT‐qPCR and immunoblotting. Results showed that the expression of SLC39A14 and E‐cadherin was increased upon silencing circ_001842 or overexpressing miR‐502‐5p (*P* < .05), while the expression of N‐cadherin and MMP‐9 was decreased (*P* < .05) (Figure 5A, B), suggesting a decrease in EMT level in Caki‐1 cells. After silencing circ_001842 or overexpressing miR‐502‐5p, the migration and invasion abilities of Caki‐1 cells were reduced (*P* < .05, Figure 5C‐F). To investigate whether circ_001842 and miR‐502‐5p regulated the EMT level in RCC cells via SLC39A14, the cells were transfected with oe‐SLC39A14 after silencing circ_001842 or overexpressing miR‐502‐5p. RT‐qPCR and immunoblotting showed that the expression of E‐cadherin was decreased after SLC39A14 overexpression (*P* < .05), while the expression of SLC39A14, N‐cadherin and MMP‐9 was elevated as compared with the cells treated with single plasmid (*P* < .05, Figure 5A and B). In addition, cell migration and invasion were enhanced following SLC39A14 overexpression (*P* < .05, Figure 5C‐F). The aforementioned data supported that silencing circ_001842 or overexpression of miR‐502‐5p and subsequent overexpression of SLC39A14 could restore the EMT level of Caki‐1 cells to some extent. It was demonstrated that circ_001842 silencing could impair RCC cell proliferation, migration, invasion and EMT via miR‐502‐5p‐mediated SLC39A14 inhibition.

### 3.6 circ_001842 silencing inhibits the growth of RCC tumours in vivo

**FIGURE 6:**
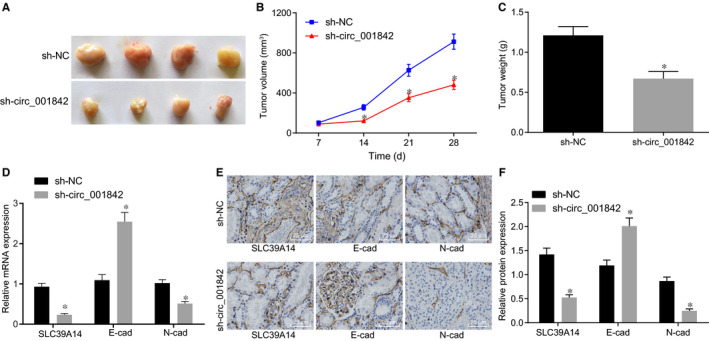
Silencing of circ_001842 curbs the progression of RCC in vivo. A, The tumour size in Caki‐1‐bearing nude mice, n = 16. B, The tumour size in Caki‐1‐bearing nude mice after different treatments, n = 16. C, The tumour weight of nude mice after 4 weeks of inoculating Caki‐1 cells, n = 7. D, The mRNA expression levels of SLC39A14, E‐cadherin and N‐cadherin in Caki‐1 cells after different treatments, determined by RT‐qPCR. E‐F, The protein expression levels of SLC39A14, E‐cadherin and N‐cadherin in Caki‐1 cells after different treatments measured by immunohistochemistry, scale bar = 25 μm. Data (mean ± SD) from three cell experiments were compared between two groups using unpaired t test and between multiple groups using two‐way analysis of variance followed by Tukey's post hoc test for panel B. n = 16, *P < .05 vs the cells treated with sh‐NC

Nude mice were injected with Caki‐1 cells transfected with sh‐NC and sh‐circ_001842, and 7 nude mice were randomly selected from each group every 7 days. Tumours were extracted and weighed, and the tumour volume was measured. Compared with the mice treated with sh‐NC, the tumorigenesis ability of the mice treated with sh‐circ_001842 treatment was lowered (n = 7, *P* < .05), and the number of tumours formed was reduced (n = 7, *P* < .05, Figure 6A‐C). The expression levels of SLC39A14, E‐cadherin and N‐cadherin were monitored by RT‐qPCR and immunohistochemistry. Results demonstrated that compared to sh‐NC treatment, expression levels of SLC39A14 and N‐cadherin were decreased while E‐cadherin expression was increased in the mice with sh‐circ_001842 (all *P* < .05), which also manifested lower cell migration ability and inhibited tumour deterioration (Figure 6D‐F). The above experimental results implied that inhibition of circ_001842 reduced the expression of SLC39A14, EMT level and tumorigenic ability in vivo.

## 4 DISCUSSION

RCC is one of the most fatal urinary cancers, and as its early symptoms are usually unnoticed, patients typically present with metastatic disease at the initial diagnosis.^21^ Despite some therapeutics like IMA901, the first therapeutic vaccine applied in RCC treatment that yielded fruitful outcome, the development of potential therapeutic strategies for RCC remains challenging.^22^, ^23^ Therefore, it is necessary to elucidate molecular mechanism underlying RCC. Circular RNAs have been found to serve as critical regulators in various human cancers.^24^ The role of circ_001842 in RCC is currently unclear. Hence, our study explored how circ_001842 impacted tumorigenesis in RCC. We discovered that silencing circ_001842 suppressed the development of RCC by disturbing miR‐502‐5p‐mediated inhibition of SLC39A14.

Firstly, our study has provided evidence that circ_001842 expression is increased in RCC tissues and cells, and down‐regulation of circ_001842 inhibits RCC cell proliferation, migration and invasion abilities. In a similar finding, a recent study found high expression of circ‐ZNF609 in RCC cell lines, which accelerate cancer cell invasion and proliferation.^11^ circ_101882 is also found overexpressed in gastric cancer tissues and cell lines, while poorly expressed circ_101882 has an antitumour effect, inhibiting cancer cell growth.^25^ Another investigation revealed a high expression of circABCC2 in hepatocellular cancer, while a reduction of circABCC2 represses hepatocellular cancer cell invasion and proliferation, but advances apoptosis,^26^ a finding that was similar to our result. Our findings verified that circ_001842 promoted tumorigenic ability and lymph node metastasis in vivo. Taken together, our findings provide evidence for the first time that circ_001842 is an oncogenic non‐coding RNA.

In addition, we provided evidence that circ_001842 upgraded RCC cell proliferation and metastasis, specifically by increasing SLC39A14 expression. SLC39A14 has been found as involved in tumorigenesis of several cancers and has been identified as a novel biomarker for a variety of cancers,^19^ which is in agreement with our finding. SLC39A14 is known to be up‐regulated in gastric cancer.^18^ Another recent study demonstrated that the expression level of SLC39A14 is amplified in hepatocellular carcinoma cells and tissues.^27^ Aligned with these studies, our study revealed that a high expression of SLC39A14 advanced the evolution of RCC. Moreover, miR‐502‐5p induced SLC39A14 expression and thus promoted RCC cell proliferation and metastasis. In sum, overexpressed circ_001842 promoted the biological function of RCC cells via the up‐regulation of SLC39A14 by binding to miR‐502‐5p. The marker E‐cadherin, in particular, has been related to lymph node metastasis in laryngeal carcinoma.^28^ A major implication of our findings is that circ_001842 promotes tumorigenic ability and lymph node metastasis in vivo.

**FIGURE 7:**
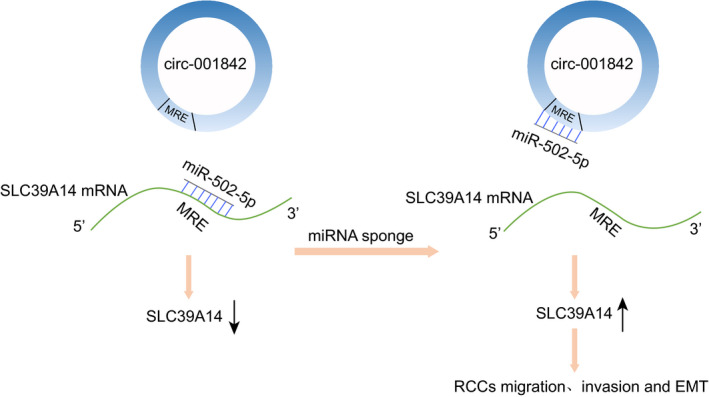
A mechanistic map depicting the role of circ_001842 in RCC. circ_001842 increases the expression of SLC39A14 by decaying miR‐502‐5p, thereby promoting the invasion, metastasis and inflammation of RCC cells

In conclusion, we ascertained the carcinogenic role of circ_001842 in RCC and found circ_001842 increased the expression of SLC39A14 by competitively binding to miR‐502‐5p, thereby promoting invasion, metastasis and inflammation of RCC cells (Figure 7). Therefore, the circ_001842/miR‐502‐5p/SLC39A14 axis may be considered as a new biomarker for prognosis and a promising therapeutic target for RCC treatment. Nonetheless, the current study only presents a theoretical basis for the mechanism of circ_001842 involvement in RCC. Further research is required to explore the specific mechanisms involving various other molecular pathways involved in RCC carcinogenesis.

## CONFLICT OF INTEREST

The authors declare that they have no conflict of interest.

## AUTHOR CONTRIBUTIONS

JWZ and JFF conceived the study; JWZ, YDW and YWY curated the data; QF, YML and JFF performed formal analysis; JWZ and YWY participated in investigation; QF contributed to methodology; YDW collected resources; GX developed the software; JFF supervised the study; YML contributed to validation; QF and GX contributed to visualization; JWZ, QF, YDW, GX and YML wrote the original draft of the manuscript; and YWY and JFF wrote, reviewed and edited the manuscript. Jiawei Zeng: Conceptualization (equal); Data curation (equal); Investigation (equal); Writing‐original draft (equal). Qian Feng: Formal analysis (equal); Methodology (equal); Visualization (equal); Writing‐original draft (equal). Yaodong Wang: Data curation (equal); Resources (equal); Writing‐original draft (equal). Gang Xie: Software (equal); Visualization (equal); Writing‐original draft (equal). Yuanmeng Li: Formal analysis (equal); Validation (equal); Writing‐original draft (equal). Yuwei Yang: Data curation (equal); Investigation (equal); Writing‐review & editing (equal). Jiafu Feng: Conceptualization (equal); Formal analysis (equal); Supervision (equal); Writing‐review & editing (equal).

## Supporting information

Fig S1

Fig S2

## Data Availability

The data sets generated and/or analysed during the current study are available from the corresponding author upon reasonable request.
